# Down Syndrome Biobank Consortium: A perspective

**DOI:** 10.1002/alz.13692

**Published:** 2024-01-25

**Authors:** Iban Aldecoa, Isabel Barroeta, Steven L. Carroll, Juan Fortea, Anah Gilmore, Stephen D. Ginsberg, Samuel J. Guzman, Eric D. Hamlett, Elizabeth Head, Sylvia E. Perez, Huntington Potter, Laura Molina‐Porcel, Ruma Raha‐Chowdhury, Thomas Wisniewski, William H. Yong, Shahid Zaman, Sujay Ghosh, Elliott J. Mufson, Ann‐Charlotte Granholm

**Affiliations:** ^1^ Pathology Department Hospital Clinic de Barcelona‐University of Barcelona Barcelona Spain; ^2^ Neurological Tissue Bank of the Biobank Hospital Clinic de Barcelona‐FCRB/IDIBAPS Barcelona Spain; ^3^ Neurology Department Hospital de la Santa Creu i Sant Pau, Neurology Barcelona Spain; ^4^ Department of Pathology & Laboratory Medicine Medical University of South Carolina Charleston South Carolina USA; ^5^ University of Colorado Denver Anschutz Medical Campus, Neurosurgery Aurora Colorado USA; ^6^ Center for Dementia Research, Nathan Kline Institute Orangeburg New York USA; ^7^ Departments of Psychiatry Neuroscience & Physiology, and the NYU Neuroscience Institute, New York University Grossman School of Medicine New York New York USA; ^8^ Department of Pathology University of Colorado Anschutz Medical Campus Aurora Colorado USA; ^9^ Department of Pathology and Laboratory Medicine University of California Irvine, UCI School of Medicine D440 Medical Sciences I Irvine California USA; ^10^ Barrow Neurological Institute Translational Neurosciences and Neurology Phoenix Arizona USA; ^11^ University of Colorado Denver Anschutz Medical Campus, Neurology Aurora Colorado USA; ^12^ Alzheimer's Disease and Other Cognitive Disorders Unit Neurology Service, Hospital Clínic, IDIBAPS, University of Barcelona Barcelona Spain; ^13^ Department of Psychiatry Cambridge Intellectual & Developmental Disabilities Research Group University of Cambridge Cambridge UK; ^14^ Center for Cognitive Neurology, Departments of Neurology, Pathology and Psychiatry New York University Grossman School of Medicine New York New York USA; ^15^ Department of Zoology Cytogenetics and Genomics Research Unit Kolkata India

**Keywords:** Alzheimer's disease, biobanking, brain banking, Down syndrome, repository, research

## Abstract

Individuals with Down syndrome (DS) have a partial or complete trisomy of chromosome 21, resulting in an increased risk for early‐onset Alzheimer's disease (AD)‐type dementia by early midlife. Despite ongoing clinical trials to treat late‐onset AD, individuals with DS are often excluded. Furthermore, timely diagnosis or management is often not available. Of the genetic causes of AD, people with DS represent the largest cohort. Currently, there is a knowledge gap regarding the underlying neurobiological mechanisms of DS‐related AD (DS‐AD), partly due to limited access to well‐characterized brain tissue and biomaterials for research. To address this challenge, we created an international consortium of brain banks focused on collecting and disseminating brain tissue from persons with DS throughout their lifespan, named the Down Syndrome Biobank Consortium (DSBC) consisting of 11 biobanking sites located in Europe, India, and the USA. This perspective describes the DSBC harmonized protocols and tissue dissemination goals.

## 1 BACKGROUND

Down syndrome (DS) or trisomy 21, a developmental genetic condition caused by a partial or entire trisomy of chromosome 21 (Chr. 21), is the most prevalent genetic cause of intellectual disability worldwide.^1^ Due to improved healthcare, the lifespan of individuals with DS has increased significantly over the last few decades. With this greater longevity, it has become apparent that individuals with DS are at greater risk for Alzheimer's disease (AD) compared to the general population,^2^, ^3^, ^4^ which is accompanied by an alarming uptick in comorbidity at younger ages (40s–50s) than is typical for late‐onset AD. Indeed, AD is now described as a limitation to any further increases in the healthy lifespan of people with DS.^5^ Trisomy 21 leads to increased risk for multiple diseases, from cardiac malformations to a higher risk of leukemia^6^, ^7^ and multiple neurological complications (such as epilepsy^8^) resulting from brain malformations.

Individuals with DS can achieve self‐sufficiency in multiple activities of daily living, resulting in an independent and fulfilling life, due to improved medical care and a greater awareness of the medical, physical, and psychological features of the condition. Despite these advances, it remains challenging to conduct studies of the cellular and molecular pathobiology of AD in DS, particularly using *postmortem* tissues obtained from well‐characterized individuals with DS and age‐matched non‐DS controls. DS‐AD neuropathology closely resembles that observed in late‐onset AD (LOAD), including an age‐dependent onset of amyloid‐β (Aβ) plaques,^9^ neurofibrillary tangles (NFTs),^10^, ^11^, ^12^ and progressive degeneration of basal forebrain cholinergic neurons (BFCNs), locus coeruleus (LC) noradrenergic neurons, hippocampal neurons, and corticocortical neuron network dysfunction.^7^ Although studies suggest that development of AD neuropathology and associated dementia symptoms are similar between individuals with LOAD and DS‐related AD, those with DS exhibit neurodegeneration at an earlier age and persistent increases in brain inflammation,^13^, ^14^, ^15^ which may contribute to the early onset of dementia in this population.

Although research into AD neuropathologic change (ADNC) is mainly amyloid and tau centric, multiple copathologies are prevalent, including features of Lewy body disease, limbic‐predominant age‐related TDP43 encephalopathy (LATE) with or without hippocampal sclerosis, aging‐related tau astrogliopathy (ARTAG), or cerebrovascular disease (CVD) in the brains of either LOAD or DS‐related AD.^16^, ^17^ Despite these complex pathological features, studies of the pathogenesis of the neurobiology underlying DS and DS‐AD using state‐of‐the‐art technologies are lacking due to the shortage of available tissue from DS cases as well as age‐matched neurotypical controls. Providing a needed biosample resource is one of the major focuses of the DSBC, which was implemented in 2017.

Findings derived from human DS‐AD research have significant translation to our understanding of LOAD, a disease that has reached epidemic proportions in the Western world.^18^ In this regard, studying AD in individuals with DS will enhance our understanding of the neurobiology of AD and potential biomarkers that predict cognitive decline and neuropathology. Currently, most brain banks are focused on collecting brain tissues from individuals with prodromal and LOAD as well as familial AD (FAD), which have provided a greater understanding of the neurobiology and genetic causes of these diseases.^19^, ^20^ However, due to the limited numbers of brain banks collecting tissues from *premortem* clinically and *postmortem* neuropathologically well‐characterized individuals with DS across the lifespan, there is a dearth of information identifying the molecular and cellular events resulting from trisomy of Chr. 21. To offset this disparity, the DSBC was founded in 2017, with the goal of enhancing DS brain donations for research. At present, the DSBC is an international network of brain banks located at 11 different performance sites. The DSBC objective is to create an international network of brain banks providing high‐quality tissue and biofluid samples of DS to researchers worldwide. The current perspective is focused on discussing the input and output parameters of the DSBC and informing researchers about its existence to enhance use of this dedicated collection of brain tissues as well as biofluids for research in DS.

## 2 METHODS

### 2.1 Overview

The DSBC currently consists of research groups at 11 different entities (Table 1). Since its inception, the DSBC developed a website and a database to view sample inventory, medical, and demographic information available in North America and Europe. The DSBC does not have a usage fee for academic researchers, although the collaborating brain banks may have local fees to cover tissue handling, storage, and shipping costs. To submit a tissue request, researchers can download the tissue request form from the DSBC website (link: https://medschool.cuanschutz.edu/neurosurgery/research‐and‐innovation/services/down‐syndrome‐biobank) and email the tissue request form to any of the investigators listed on the website. Each member of the DSBC reviews the request for the quality of the science, available funding support, and which sites can contribute to the request.

**TABLE 1 alz13692-tbl-0001:** Components of DSBC consortium and specific roles.

Investigator	Role	Institution
Ann‐Charlotte (“Lotta”) Granholm	Coordinator	CU Anschutz, USA, and Karolinska Institutet, Stockholm, Sweden
Elliott J. Mufson	Co‐Principal Investigator	Barrow Neurological Institute, USA
Elizabeth Head	Co‐Principal Investigator	UC Irvine, USA
William Yong	Neuropathology	UC Irvine, USA
Huntington Potter	Collaborator	CU Anschutz, USA
Jennifer Eschbacher	Neuropathology	Chair, Pathology, Barrow Neurological Institute, USA
Juan Fortea Isabel Barroeta	Neurology	Hospital Sant Pau, Barcelona, Spain
Iban Aldecoa Laura Molina‐Porcel	Neuropathology/Neurology	Biobank‐FRCB‐IDIBAPS, Hospital Clinic Barcelona, Spain
Steve Carroll	Neuropathology	Chair, Pathology Dept., MUSC, USA
Thomas Wisniewski	Neuropathology/Neurology	NYU Grossman School of Medicine (NYUGSOM), USA
Stephen D. Ginsberg	Collaborator	Nathan Kline Institute (NKI)/NYUGSOM, USA
Shahid Zaman	Neurology	Cambridge University, UK
Ruma Raha‐Chowdhury	Neuropathology/Neurology	Cambridge University, UK
Andre Strydom	Neurology/Geriatrics	London Down Syndrome Consortium (LonDownS), UK
Samuel J. Guzman	Neuropathology	CU Anschutz, USA
Sujay Ghosh	Neurology	University of Calcutta, India

Green‐shaded lines are neuropathologists.

Before the formation of the DSBC in the USA, cohorts located across sites (Table 1) used different protocols for brain procurement and individually could not generate enough samples for well‐powered studies. Moreover, sites not associated with an Alzheimer's Disease Research Center (ADRC) did not have access to age‐matched controls or LOAD/FAD cases – an essential set of controls for rigorous research studies. The creation of the consortium also provides researchers access to AD, FAD, and control cases from shared resources.

DSBC members acknowledged that harmonized protocols for brain procurement and tissue selection would be difficult to fully implement across all collaborating brain banks and perhaps hinder the inclusion of future collaborating sites that already have established protocols and workflows, as well as local legal requirements. Repeated training sessions and in‐person meetings allow for the development of harmonized methods between sites. DSBC holds a pragmatic approach seeking equilibrium between the minimal and ideal tissue samples obtained for frozen storage and paraformaldehyde/formalin‐fixed paraffin‐embedded (FFPE) blocks and sections, as well as neuropathological diagnostic criteria according to published protocols.^21^ The overall goal is to outline a minimum of sample testing and assessments to standardize the quality of tissues and biofluids between sites. The DSBC steering committee, which consists of one PI per site, is responsible for implementing the appropriate quality assurance activities and measures at each tissue repository. New sites can apply for membership in the consortium by submitting a request to the steering committee, and new members are discussed at the next (bimonthly) meeting of this committee and voted on.

### 2.2 Tissue and biofluid processing

All DSBC donors must donate at least the brain, which is a requirement for a donor to be registered in the DSBC database. If allowed by the local brain bank and approved by the donor's family, the site can obtain spinal cord, cerebrospinal fluid (CSF), vitreous and aqueous humor, eyes, inner ear, and *postmortem* blood. Blood is processed for serum, plasma and buffy coat. Lumbar and/or cranial CSF (25 mL) is obtained when feasible) CSF and blood tubes are placed on ice and transported to the site laboratory. The serum tubes are placed on ice for half an hour, after which they are spun at 4°C for 20 min and aliquoted into 0.5 mL aliquots. The plasma samples are immediately spun at 4°C for 20 min and aliquoted into 0.5 mL Eppendorf tubes. CSF samples are centrifuged at 4000 × *g* for 10 min at 4°C and then aliquoted into 0.5 to 0.6 mL Eppendorf tubes. The aqueous and vitreous fluids are collected directly into 0.5 to 0.6 mL Eppendorf tubes and all fluids are frozen immediately at −80°C. CSF and blood are not collected in cases with a *postmortem* interval (PMI) above 12 h.

Brains are photographed from multiple directions and sliced into 1 cm coronal slices using a precision Plexiglass brain jig printed using a 3D printer to aid in the collection of uniform slices (Figure S1). The coronal slices are placed on a cutting board and photographed prior to microdissection for freezing (left hemisphere) and fixation in paraformaldehyde or formaldehyde (right hemisphere). Personnel at each site have been trained by neuropathologists in brain procurement procedures and dissection techniques, so that each brain is handled in a similar fashion.

Paraffin embedded sections are stained with hematoxylin and eosin (H&E) and Bielschowsky silver staining according to National Institute on Aging (NIA)‐Alzheimer's Association (AA) protocols.^21^ Immunohistochemical stains including alpha‐synuclein, amyloid‐beta, phospho‐tau, and TDP43 or phosphorylated TDP43 are performed to enable an adequate neuropathological examination. Additional stains such as three‐repeat tau, four‐repeat tau, alpha B‐crystallin, alpha‐internexin, Fused in Sarcoma (FUS), C9RANT, or other immunostains are considered on a case‐by‐case basis.^22^

**RESEARCH IN CONTEXT**

1. **Systematic reviewe**: Prior to the formation of the Down Syndrome Biobank Consortium (DSBC), there was no concerted effort to collect and disseminate brain tissue and associated fluids from persons with Down syndrome and appropriate controls.
2. **Interpretation**: As evidenced by the many successful tissue requests and publications resulting from the work in DSBC and its consortium members, this biobank has been able to provide an international research network focused on providing high‐quality samples to researchers across the world.
3. **Future directions**: Continued funding of this or similar research consortia focused on *postmortem* studies of brain tissue from individuals with Down syndrome will  enhance research and may lead to the development of novel treatment paradigms for persons with Down syndrome and Alzheimer's disease or other neurodegenerative conditions.

A neuropathology report is produced following[Table alz13692-tbl-0001] a modified NIA‐AA protocol adjusted to suit the slightly different neuropathology observed in those with DS.^21^ In this regard, the DSBC requires the sampling and evaluation of a minimum of 16 brain regions (Table 2A). Although it is less than the 19 brain regions recommended by the NIA‐AA standardized neuropathology protocol,^21^ these were selected to ensure anatomical uniformity between the different collaborating sites. Nevertheless, the DSBC recommends that the consortium members sample an additional 15 brain regions, if possible (Table 2B). Based on local resources, all participating sites may not be able to collect all areas described in Table 2.

**TABLE 2:** A: Minimum areas for FFPE sampling in DSBC cases and data report.

A. Minimal amount of brain regions
Superior frontal gyrus/midfrontal gyrus (BA9/46)
Pre‐ and postcentral gyri/primary motor cortex (BA4)
Anterior cingulum – corpus callosum/BA24
Lenticular at level of nucleus basalis of Meynert (NBM)
Anterior thalamus – with mammillary body
Ventromedial thalamus – with subthalamic nucleus
Hippocampus + parahippocampal gyrus (at level of lateral geniculate nucleus)
Mid‐low temporal gyri and amygdala/BA28
Calcarine gyrus/BA17
Substantia nigra – midbrain
Pons with Locus coeruleus
Medulla oblongata
Cerebellar vermis
Cerebellar hemisphere + dentate nucleus
Upper and medial temporal gyri/BA21
Low parietal gyrus/angular gyrus/BA39/40

B. Additional recommended areas
White matter semi‐oval frontal center
White matter semi‐oval occipital center
Orbitofrontal cortex
Temporal pole
Posterior cingulate (BA23/31) and precuneus (BA7)
Head of caudate nucleus + accumbens nucleus
Dorsal thalamus with lateral geniculate nucleus
Anterior insula
Anterior hippocampus with entorhinal cortex
Lower pons
Spinal cord, cervical, dorsal, lumbar, sacral
Optic chiasm
Olfactory bulb (especially for COVID cases)
Pituitary gland
Pineal gland
Choroid plexus
Eyes/inner ears

A neuropathology report is generated locally for each case and discussed at bimonthly clinicopathological conferences (CPCs) with consortium members. An important goal for the DSBC is to generate a unified neuropathological staging system for brains with DS‐AD, like that for LOAD^23^, ^24^ but also to educate researchers and trainees regarding a standardized staging procedure for DS‐AD. The neuropathological diagnosis and staging criteria have been harmonized between the collaborating centers (see Supplementary Data 2). The neuropathology report and representative scanned slides will be available to researchers once a research protocol is approved by an Institutional Review Board (IRB).

#### 2.2.1 Consent process

Participants are recruited from institutionalized and noninstitutionalized individuals with DS in the USA, Europe, and India. The international span of this consortium is unique; we now have six sites located in five states in the USA, four in Europe and a site in Calcutta, India (Table 1). Recruitment of participants occurs via each participating program, monitored by each site's IRB or ethics committee with consent and assent obtained on an individual basis, which will be maintained at participating sites to maintain privacy.

#### 2.2.2 Cognitive assessment

Criteria for the diagnosis of dementia in DS are challenging against the background of pre‐existing intellectual impairment but include informant‐based and direct measures.^6^, ^25^, ^26^, ^27^ We recognize that each site has unique and overlapping test batteries, and thus we will work to identify a minimal unified dataset that can be shared (similar to the National Alzheimer's Coordinating Center [NACC], Uniform Data Set [UDS]^28^). Since 2020, NACC has had a module for adults with DS (https://naccdata.org/data‐collection/forms‐documentation/dsm). Most of the clinical sites included in the consortium are members of one of the clinical DS networks, including ABC‐DS, Life‐DSR or Horizon 21, allowing harmonized protocols for cognitive data at least within these networks. In Europe, all sites use a modified version of the CAMDEX, a standardized instrument that has been translated into several languages.^29^ The Cambridge Examination for Mental Disorders of Older People with Down Syndrome and others with intellectual disabilities (CAMDEX‐DS‐II) is a second edition of a validated assessment for the diagnosis of dementia in people with DS and others with intellectual disabilities.^29^

#### 2.2.3 Genetic screening and karyotyping

Confirmation of trisomy 21 is obtained from the medical records and/or from karyotyping at each site. Each site performs apolipoprotein E (APOE) genotyping and other genetic testing in accordance with their individual IRBs. DS diagnosis can also be confirmed using Western blots or other measures for overexpression of Chr. 21‐specific gene products such as the superoxide dismutase 1 (SOD1) and amyloid precursor protein (APP) genes.

#### 2.2.4 Tissue request committee

A tissue request committee was formed that consists of one PI from each site. This committee has monthly meetings, or as soon as a new tissue request is received. We have used a hybrid approach for neuropathology and brain bank coordination; histopathology, tissue requests, and training of IRB issues are sometimes site‐specific due to the differences between IRB requirements between Europe and USA. Typically, tissue requests come from several sites based on the availability of samples. For example, the Medical University of South Carolina site has access to more than 325 cases, with over 40 control cases that match in age with University of California, Irvine or Fundació de Recerca Clínic Barcelona‐Institut d'Investigacions Biomèdiques August Pi i Sunyer (FRCB‐IDIBAPS)  DS cases and many early‐onset AD (EOAD) or LOAD cases in a wide age range. A major remaining issue is the lack of access to tissue from younger donors, since AD pathology begins early in life in those with DS.^10^ A possible way of addressing this caveat would be to engage families or caregivers of young individuals with DS that are treated at various children's hospitals to create a large registry of donors of all ages. For example, the Phoenix DSBC has been in discussions with staff and physicians that treat ∼300 young people with DS from birth to 18 years of age at the Phoenix Children's Hospital. A close collaboration with the medical examiner's (MEs) office and/or children's hospitals at some sites might allow inclusion of cases from younger individuals with DS.

Each collaborating brain bank must follow local guidelines and legal frameworks. In this sense, a tissue request to the DSBC consortium may be divided into separate requests to each brain bank based on the availability of samples at each site and provide support in the management of the tissue requests, if needed. This process has worked well in the last 4 years thanks to the harmonized processing protocols among sites, resulting in more than 30 fulfilled tissue requests in the USA and Europe.

#### 2.2.5 Sample storage and distribution

Frozen or fixed biofluids and tissues are stored at each DSBC brain repository site. Following a consensus medical file review and neuropathological staging, de‐identified information regarding each brain is entered into a multisite REDCap database. Data linked to each brain donation will be available to researchers when they have demonstrated a valid IRB and have received the committee's approval for tissue distribution. Anonymity is strictly maintained at each of the sites by local honest brokers and PIs. All cases are de‐identified by a Global Unified ID number (GUID).

## 3 RESULTS

### 3.1 Consortium input

The first objective of the DSBC is to develop an international repository for the collection and distribution of *postmortem* DS brain, serum, plasma, and CSF (*input objective*) across the entire lifespan to investigate the neurobiological mechanisms and generate novel biomarkers for AD in DS. In the field of DS research there is a critical need to combine several clinical DS cohorts and brain banks to generate enough well‐characterized DS brain donations with low PMIs and high‐quality clinical and biomarker data.

This unique consortium has expanded to include 11 research groups, currently all highly productive and well‐known investigators in the AD and DS field (Table 1). Dr. Ann‐Charlotte Granholm (CU Anschutz) serves as the contact PI and assumes overall fiscal and administrative management. Dr. Elliott J. Mufson (Barrow Neurological Institute) and Dr. Elizabeth Head (UC Irvine) serve as co‐PIs on the project and render continuous support for the consortium and resulting studies. Recently, six new brain banks/research groups were added to the consortium: Dr. Thomas Wisniewski (NYUGSOM), Dr. Stephen D. Ginsberg (NKI/NYUGSOM), Dr. Shahid Zaman and Dr. Ruma Raha–Chowdhury (Cambridge University, UK), Dr. Andre Strydom (King's College, London, UK) and Dr. Sujay Ghosh (University of Calcutta, India).

During the first 4 years of the consortium, we performed training sessions to harmonize tissue collection protocols, developed tissue request forms, and developed a standard operating procedure (SOP). As shown in Table 1, there are eight dedicated neuropathologists divided between the USA and Europe who orchestrate the dissecting and staging of brains. The final neuropathological diagnosis along with representative images will be uploaded to the REDCap database housed at the University of Colorado (CU). We also distribute to all sites a unified brain‐cutting apparatus (a “brain jig”) that is novel to the field and was produced via a 3D printer at the University of Denver (Figure S1). These brain jigs are used to generate close to identical coronal slices of equal thickness at all sites to normalize the brain regions dissected for diagnosis and research and to harmonize the process among sites.

To date, thirty brain donations have been received via the DSBC network. In addition to these DSBC‐generated donations, collectively the consortiuum brain banks included i have access to more than 300 DS or DS‐AD donated brains, as well as a large numbers of AD, FAD and age‐matched non‐DS controls (Table 3).

**TABLE 3:** Available cases in DSBC member brain banks.

	DS‐AD	DS‐nondemented	Partial trisomy	DS < 18 years	Controls	Controls < 18 years	Total cases
No. cases	94	25	1	21	144	19	304
Average PMI	14.6	14.3	4.9	29.1	14.5	17.1	
PMI range	5 to 30	7.75 to 24.5		26 to 32	4.3 to 30	12 to 22	
Female %	45	40		48	47	63	

### 3.2 Consortium output

A secondary main objective is to promote the use of DSBC samples for research (*output objective*). The DSBC has no active funding for research but tries to fulfill this goal in several ways. Each DSBC member is an active researcher in the DS field. DSBC membership gives access to valuable information on samples available in the collaborating brain banks, shared knowledge regarding the suitability of these samples for different research purposes, and neuropathological and neuroscience expertise in DS. Furthermore, the DSBC makes known to the research community the existence of these samples and promotes its use to external researchers by providing support for the delivery of adequate samples, demographic, genotype, and clinical information needed for research projects. The DSBC website (see earlier discussion) contains information about the cases, brochures in several languages to inform families about the process (Supplemental dataset 3), tissue request forms, and other forms to simplify the donation process. Recently, a working group within the International Trisomy 21 Research Society (T21 RS), known as the Neuropathology Working Group, was formed to disseminate information regarding brain tissue availability to the international research community s in the DS field.

Since its founding, the DSBC has filled more than 30 tissue requests from researchers who are part of the consortium, but also from out‐of‐network researchers located in North America and Europe. It is expected that more tissue requests will be submitted and filled by the consortium as it becomes more well known and the number of donations increases. An important activity of the consortium is the Clinical Pathologic Case Presentations (CPCs) that occur bimonthly, where —between one and three DSBC cases are discussed and staged.

Another important output of the consortium is publications and successful grant applications. In the first 5 years of the consortium, DSBC investigators generated preliminary data for new grant submissions and published findings in more than 35 publications (e.g.,^2^, ^6^, ^7^, ^27^, ^30^, ^31^, ^32^, ^33^, ^34^, ^35^, ^36^, ^37^, ^38^, ^39^, ^40^, ^41^, ^42^, ^43^, ^44^). During the pandemic, we published special issues in journals focused on the neurobiology of DS as a consortium, which appeared in the *Journal of Clinical Medicine* (14 articles; https://www.mdpi.com/journal/jcm/special_issues/Down Syndrome_Aging), *Frontiers in Aging Neurosciences* (11 articles; https://www.frontiersin.org/research‐topics/15748/down‐syndrome‐neurodegeneration‐and‐dementia) and participated in a book entitled “*The Neurobiology of Aging and Alzheimer's Disease in Down Syndrome*”. Overall, access to well‐characterized DS brain tissue increased the productivity of the DSBC research teams.

The activities in the DSBC, together with those in existing as well as newly formed clinical networks in the USA [e.g., ABC (amyloid plaques (A), NFT stage (B), and neuritic plaque score (C))‐DS, Life‐DSR] and Europe (Horizon 21), led to an increased awareness of clinical treatment and biological mechanisms for DS‐AD and increased National Institutes of Health (NIH) funding in this area.^6^, ^30^ Several consortium members have received NIH funding, leading to more than $15 million in funding beyond the BrightFocus grant awarded to the consortium to initiate the DSBC. Since no single clinic or brain bank has access to enough samples across all ages to provide sufficient samples to other researchers, continued funding of the DSBC network is needed to maintain its key function as a major resource for brain tissue in the DS field.

#### 3.2.1 Neurobiological findings

**FIGURE 1:**
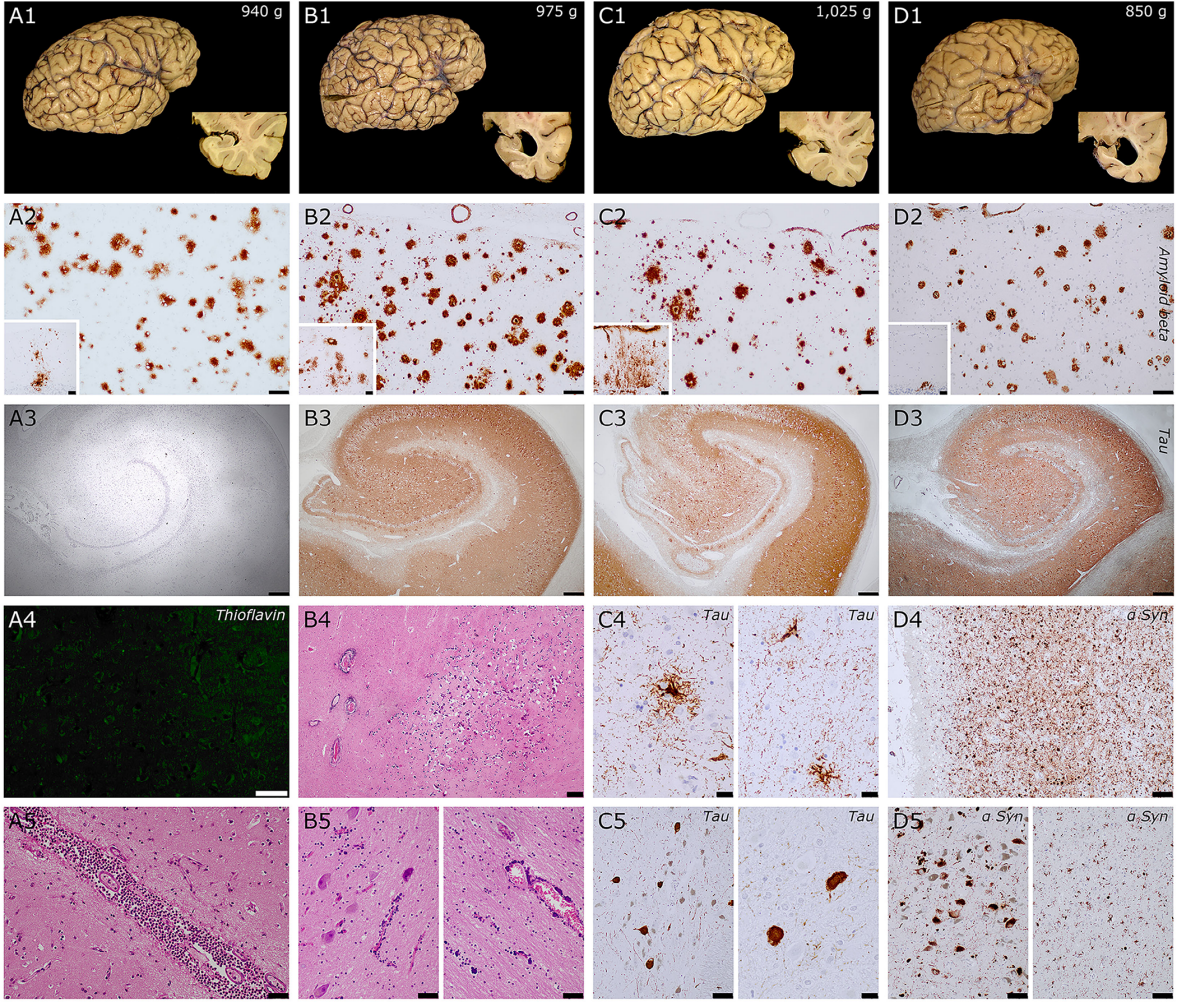
Neuropathological features of four donors with DS. A1‐5 is a female in her mid 20s, B1‐5 a male in his mid 40s, C1‐5 a male over 70 years old, and D1‐5 a female in her mid 60s. DS donors B and D had an additional clinical diagnosis of Alzheimer's disease (AD). *Similarities*: First row: macroscopic features: all cases had low brain weight, regardless of neuropathological processes; donors with concomitant AD (B and D) had more severe hippocampal atrophy. Second row: all donors had abundant Aβ in the frontal cortex that extended to cerebellum (insets) in all cases, compatible with a Thal phase 5/5. Third row: A, younger donor, lacking tau pathology, while the three other donors had extensive neurofibrillary pathology in hippocampal complex (Braak stages IV, C3) ) and V/VI ( B3 and D3). *Unique features*. In donor A, despite extensive Aβ deposits, the case was almost devoid of neuritic plaques (A4, negative thioflavin staining in neocortex). This case also had acute meningitis, that was related to the final pathological processes associated with death (bottom row, A5, pericentral cortex). Case B had extensive calcium deposits in basal ganglia (B4, globus pallidum), dentate nucleus of the cerebellum (B5, bottom row), and adjacent white matter (B5), arteriolar walls and in pericapillary areas. This pattern is reminiscent of Fahr's syndrome, that was previously reported in cases with DS. Case C had an additional tauopathy compatible with progressive supranuclear palsy, with tufted astrocytes in motor cortex (C4 left) and putamen (C4 right) and neurofibrillary tangles in substantia nigra (C5 left), subthalamic nucleus (C5 right panel), globus pallidus. and additional features such as coiled bodies (not shown). Case D also had an extensive Lewy pathology, with severe involvement of the limbic system (D4, amygdala), brainstem (D5 left, substantia nigra) neocortex (D5 right, frontal cortex). Scale bars: A3 to D3 = 500 μm; B4 = 200 μm; A2 to D2, C5 left, D4, D5 = 100 μm; A2 to D2 insets, A4, A5, B5 = 50 μm; C4, C5 right = 20 μm.

**FIGURE 2:**
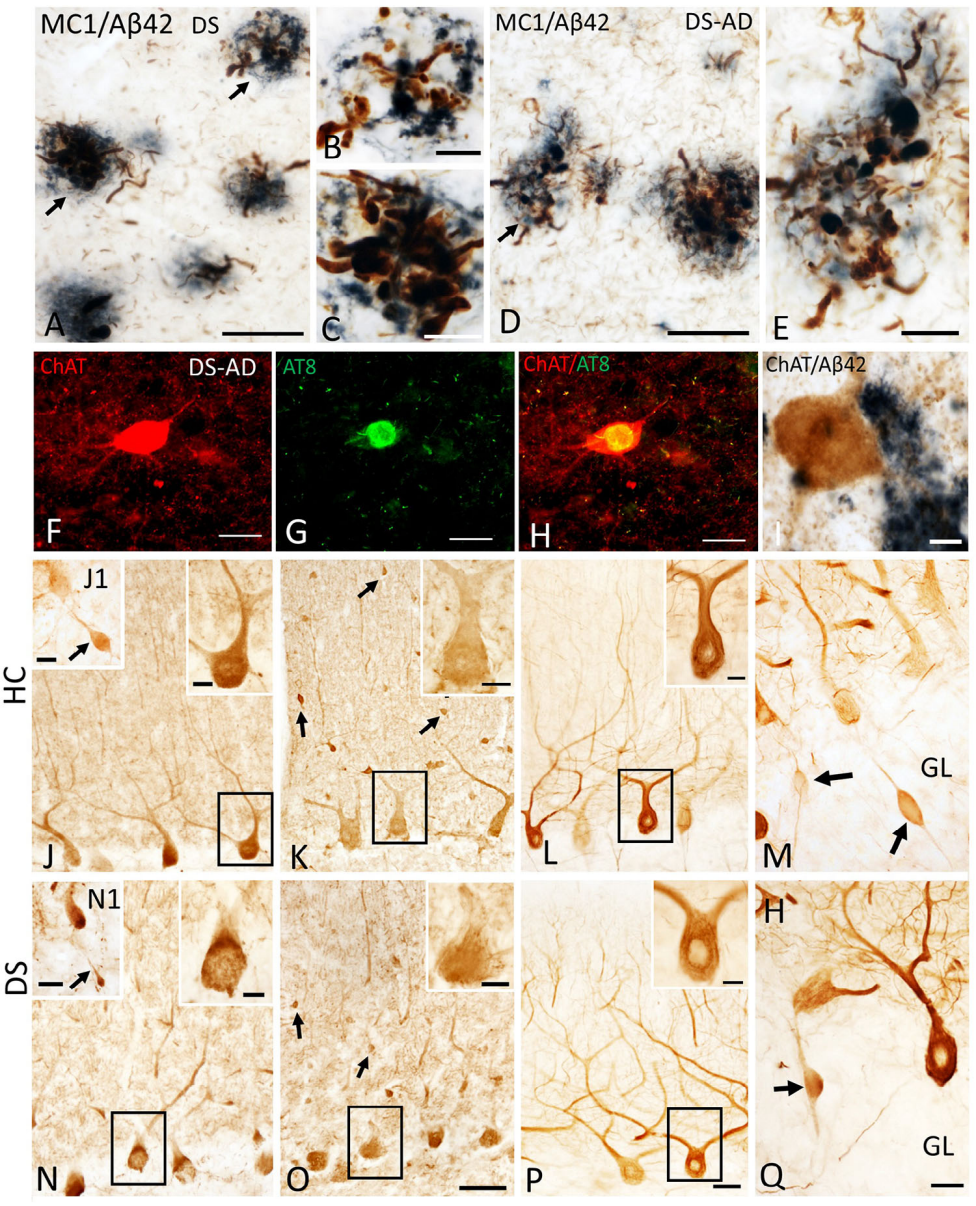
Photomicrographs of dual labeled frontal cortex sections showing dystrophic neurites displaying immunoreactivity for tau conformational epitope MC1 (brown) intermingled within Aβ42‐ir plaques (blue) in a 47‐year‐old female nondemented (A) and a 46‐year‐old male demented (D) individual with DS. Note the presence of numerous MC1 immunoreactive (‐ir) neuropile threads in demented compared to nondemented DS case. (B, C, and E) High‐power images showing bulbous nature of dystrophic neurites within Aβ42‐ir plaques from panels A and D (arrows), respectively. (F–H) Single immunofluorescence images showing normal appearing striatal choline acetyltransferase (ChAT) positive neuron (red, F), AT8‐positive NFT (green; G) in a 46‐year‐old male demented case. (H) Merged image of ChAT and AT8 immunostaining shown in F and G.  Note the intact appearance of the cholinergic striatal neuron (red) despite the presence of an AT8 reactivity (yellow) within the perikarya in this demented DS case. (I) Intact ChAT‐positive putaminal neuron (brown) despite its proximity to Aβ42 staining (blue‐black) in a 46‐year‐old male donor with DS and dementia. (J, N) Photomicrographs showing Calb‐ir Purkinje cells (PCs) in a female 66‐year‐old healthy control (HC) (J) and a female 47‐year‐old nondemented DS (N) case. Upper right insets show high‐power image of black‐boxed Calb‐ir PCs in panels J and N.  Insets J1 and N1 show cerebellar granular layer (GL) Calb‐ir axonal torpedoes (arrows) in a male 51‐year‐old healthy control (HC) and a female nondemented 60‐year‐old DS case. (K, O) Images showing Parv‐ir PCs and Parv‐ir interneurons (black arrows) within the cerebellar molecular layer (ML) in a female 69‐year‐old HC (K) and a female 44‐year‐old DS without dementia (O). Upper right insets (K, O) are higher‐magnification images of the Parv‐ir PCs shown in the black boxes. (L, P). Photomicrographs of nonphosphorylated high‐molecular‐weight neurofilaments (SMI‐32‐ir) PC dendritic arbors and axons in a female 69‐year‐old HC (L) and a male 46‐year‐old DS‐AD (P) case. Insets in L and P show high‐power images of boxed SMI‐32‐ir PCs and proximal dendrites. (M, Q) Swollen SMI‐32‐ir proximal PC axons or torpedoes (arrows) in GL of male 51‐year‐old HC (M) and female 60‐year‐old DS (Q). Scale bars:  A, D, F–H = 50 μm; B, C, E, I and insets in J, K, L, N, O, P = 10 μm; J1 and N1 insets = 30 μm; O = 50 μm and applies to J, K, M; P = 50 μm and applies to L; Q = 25 μm and applies to M.

The DSBC has provided samples for unique studies on the neuropathology of DS throughout the lifespan. Recent studies have highlighted neuropathological heterogeneity in individuals with DS,^16^, ^17^ seen in DSBC cases (Figure 1A1, B1, C1, D1)). During CPCs, the degree and types of pathology found in each case are discussed and entered into the database and website for educational purposes. The high quality of the tissue provided by the DSBC for research results in exquisite images of the neuropathobiology features examined beyond standard H&E histological methods. Figure 2 shows examples of single or dual immunostaining using antibodies against choline acetyltransferase (ChAT), tau epitopes (phosphorylated AT8 and conformational MC1 tau epitopes), neurofilaments (SMI‐32), and calcium binding proteins calbindin (Calb) and parvalbumin (Parv) visualized using the chromogen 3,3′‐diaminobenzidine (DAB) or immunofluorescence of tissue obtained by the DSBC from cortex, striatum, and cerebellum.

We also performed the first‐of‐its‐kind single‐population transcriptomic analysis comparing the genetic signature of tangle‐bearing neurons between DS with and without dementia.^44^ This study provided evidence for a different genetic signature between these groups suggesting a possible biomarker strategy. In a separate set of experiments, DSBC investigators showed early and prominent tau binding in the frontal cortex of persons with DS‐AD compared to EOAD or LOAD in the general population using two different tau positron emission tomography (PET) tracers.^35^ DSBC investigators analyzed the development of different cellular phenotypes in the cerebellum, frontal cortex, and hippocampus in pre‐ and postnatal DS cases.^44^, ^45^, ^46^, ^47^ Members of the consortium published several biomarker papers^7^, ^28^, ^41^, ^48^, ^49^, ^50^ and articles in *Nature Neurology Reviews*.^27^, ^31^ The findings obtained with the support of the DSBC have been presented at several conferences and workshops (e.g., International Conference for T21 Research Society, https://www.t21rs.org). Overall, the development of the DSBC has resulted in numerous DS publications and grant funding and has provided the impetus for increased international collaborations in this underinvestigated area of research.

## 4 DISCUSSION

The creation of the DSBC to collect donated brains from individuals with DS represents a valuable resource on multiple levels: (i) the generation and maintenance of a DS‐focused brain bank enables comparisons with other genetic or EOAD and LOAD cases serving as a potential early‐onset template; (ii) the overexpression of APP in trisomy 21 may shed light on the deleterious effects of Aβ on the development of ADNC and inform future therapeutic approaches; (iii) the unique neurodegenerative features of DS may help in the development of patient‐specific treatments for a personalized medicine approach that likely will benefit individuals with DS; and (iv) a greater understanding of the pathobiology of DS‐AD will aid in diagnosis, inclusion in clinical trials, and ultimately, treatment options for dementia. Moreover, brain tissue collected by the DSBC provides unprecedented access to high‐quality tissues for scientists in both the DS and AD research space, which led to increased funding and publications related to our understanding of the neuropathobiology of DS. The DSBC tissues are available to anyone for the cost of transportation and potential local brain bank fees. A measure of the success of the DSBC has been the yearly increase in tissue requests as knowledge of the consortium has been disseminated in research publications, presentations at conferences, and via advocacy organizations. Overall, the *input* of DSBC is to collect brain tissue from clinically well‐characterized DS cases, appropriate controls, and associated biofluids, and the *output* is to distribute these samples via a centralized website and a REDCap database.

The fact that each member of the consortium is an active researcher in the fields of DS and AD has been instrumental for the success of the DSBC highlighted by the collective acquisition of more than $15 million in funding from federal agencies that supports the research value of the consortium for human brain‐based research studies. Likely many of these scientific projects would not have been possible without the tissue collectively obtained by the DSBC. An important added value of the DSBC is that many of the sites have active cohorts of adults with DS and have collected long‐term medical information, clinical assessments, and imaging data associated with the donated tissue. To ensure proper clinical evaluation, the neurologists involved in the DSBC network are highly experienced in the care of individuals with DS, especially with age‐related complications such as epilepsy, sleep disorders, visual or hearing loss, and dementia that are common in middle‐aged or aged persons with DS (e.g.,^6^, ^7^, ^8^, ^28^, ^51^, ^52^, ^53^, ^54^). Moreover, all members of the DSBC are experienced in talking with family members or caregivers who are not aware of brain donation procedures.

### 4.1 Limitations

DS is both a neurodevelopmental and neurodegenerative disorder and poses challenges across the lifespan to individual with DS, family members, or assisted living communities, making discussions about brain donation at the time of death a difficult conversation. Consortium‐wide training ensures that all clinics are comfortable approaching a potential donor and their caregiving team. Prior conversations with caretakers/family members have proven to be advantageous to the consortium's ability to collect brain tissue. Since the consortium combines resources, even a small number of cases registered at each site will result in the whole being greater than each individual component. This is especially true for our international consortium members, who have access to large donor cohorts. The infrastructure developed by the DSBC enhances its ability to obtain brain tissue and biofluids for DS research dedicated to the betterment of the DS and DS‐AD community. Further, studies by our group (e.g.,^35^), have shown that DSBC brain tissues are of high quality with low PMIs, allowing for the investigation of the neuropathobiology of individuals with DS with or without dementia. New research approaches, including spatial transcriptomics and artificial intelligence with machine learning, will allow unique in‐depth analysis of these valuable brain tissues as reported by members of the DSBC (e.g.,^55^).

In conclusion, the DSBC's primary goals are to collect brain tissue and biofluids from clinically well‐characterized DS and control cases for distribution to DS researchers, both nationally and internationally, to increase collaborative investigations. Secondary goals include to educate graduate students, postdoctoral fellows, residents, and junior faculty about the importance of examining *postmortem* brain tissue and biofluids from people with DS and DS‐AD as well as increase the quality and quantity of scientific publications in this space.

## CONFLICT OF INTEREST STATEMENT

The authors declare no conflicts of interest. Author disclosures are available in the supporting information.

## CONSENT STATEMENT

All participants in the brain donation program have given their consent or assent, and/or *postmortem* consent was obtained from the next of kin following the passing of the participant. Each site in this consortium is covered by its own site IRB regarding brain donation and banking as well as sharing with other entities.

## Supporting information

Supporting Information

Supporting Information

Supporting Information

Supporting Information
