# Supplementary figures and images for: Down Syndrome Biobank Consortium: A perspective
Source: Alzheimers Dement. 2024 Jan 25;20(3):2262–72. doi: 10.1002/alz.13692 (PMC10984425; doi:10.1002/alz.13692)

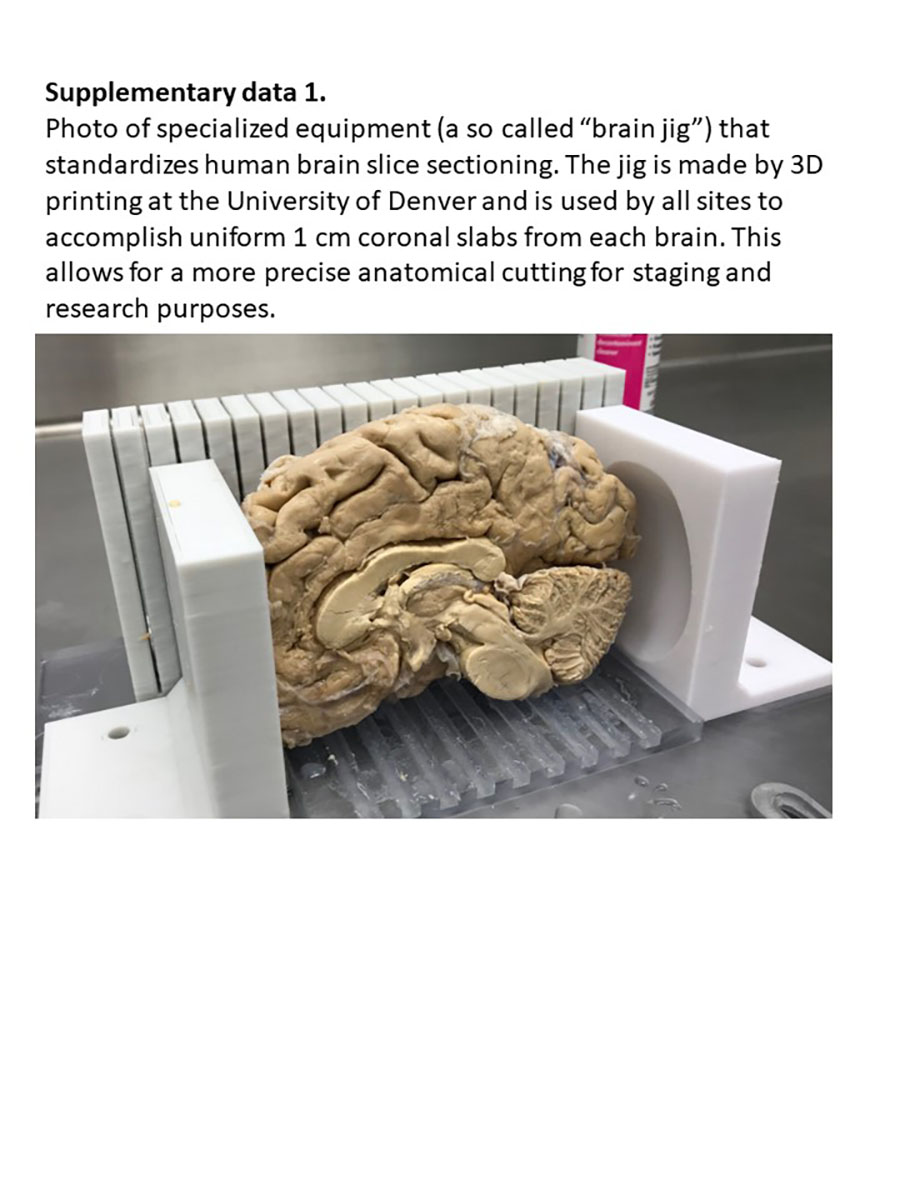

Supplement: Supplementary file 1 — Supporting Information [file ALZ-20-2262-s003.jpg]
