# Supplementary material for: Down Syndrome Biobank Consortium: A perspective
Source: Alzheimers Dement. 2024 Jan 25;20(3):2262–72. doi: 10.1002/alz.13692 (PMC10984425; doi:10.1002/alz.13692)
Supplement: Supplementary file 2 — Supporting Information [file ALZ-20-2262-s004.docx]

**Supplementary data 2**

**Supplementary data 2.**

**Main neuropathological diagnosis and staging criteria**:

- ADNC following the NIA/AA 2012 guidelines, including Thal amyloid phases, Braak neurofibrillary disease staging and CERAD neuritic plaque score.
- Cerebral amyloid angiopathy (CAA) staging and grading using the Allen types and Vonsattel grades
- Lewy body disease staging following the Braak, McKeith and 2021 consensus criteria. Alpha-synuclein would be screened at least in amygdala, olfactory bulb, substantia nigra and medulla.
- Limbic-related TDP43 encephalopathy screening and staging using the 2022 LATE update and the Josephs staging if possible. TDP43 would be screened at least in amygdala, hippocampus and frontal cortex.
- Rainwater Charitable Foundation criteria for the neuropathologic diagnosis of progressive supranuclear palsy (PSP) and Kovacs grading for PSP.
- Report of vascular lesions: descriptive report. As option, use Deramecourt et al. cerebrovascular pathology grades and VCING estimating contribution to cognitive impairment.
- Brettschneiders staging of FTLD-TDP and ALS-TDP.
- Report of any additional pathology based – when available – on consensus criteria (see e.g. Kovacs GG. Molecular pathology of neurodegenerative diseases: principles and practice. J Clin Pathol. 2019;72:725-35.).

**References for neurodegenerative disease diagnosis and staging**

1. **Alzheimer’s disease and related pathologies**

- AD NIA/AA 2012 ABC (Montine et al Acta Neuropathol (2012) 123:1–11)
- PART (Crary et al. Acta Neuropathol (2014) 128:755–766).
- Thal (Thal et al. Neurology 2002;58:1791–1800)
- Braak NFT (Braak et al. Acta Neuropathol (2006) 112:389–404)
- CERAD (Mirra et al. Neurology 1991;41:479-486)
- LATE TDP43 (Nelson et al. Acta Neuropathol. 2023 Feb;145(2):159-173.) (Josephs et al. Acta Neuropathol (2016) 131:571–585)
- 90+ contribution dementia in AD (Robinson et al. Acta Neuropathol. 2018 Sep;136(3):377-388)

1. **Vascular disease (CVD, VBI and CAA)**

- CAA Type (Allen et al. Neuropathol Appl Neurobiol. 2014 Feb;40(2):136-48)
- CAA Grade (Vonsattel et al. Ann Neurol. 1991 Nov;30(5):637-49)
- VCING estimating contribution to cognitive impairment (VCING, Skrobot et al. Brain. 2016 Nov 1;139(11):2957-2969)
- CVD and VBI Grade (Deramecourt V et al. Neurology. 2012 Apr 3; 78(14): 1043–1050)

1. **Lewy body diseases**

- PD (Braak et al. Neurobiol Aging 24 (2003) 197–211)(Alafuzoff et al. Acta Neuropathol (2009) 117:635–652. BrainNet)(Attems et al. Acta Neuropathol. 2021 Feb;141(2):159-172)
- DLB (McKeith et al., Neurology. 2017 Jul 4;89(1):88-100)

1. **ALS and FTLD TDP and Tau**

- ALS TDP43 staging (Brettschneider et al. Ann Neurol. 2013 Jul;74(1):20-38)
- FLTD-TDP subtypes (Mackenzie et al. Acta Neuropathol (2011) 122:111–113)
- FLTD behavioral variant TDP43 staging (Brettschneider et al. Acta Neuropathol (2014) 127:423–439)
- FTD MAPT mutations (Ghetti B et al. Neuropathol Appl Neurobiol. 2015 Feb;41(1):24-46)
- PSP minimum criteria (Roemer et al. Acta Neuropathol. 2022 Oct;144(4):603-614)
- PSP tau staging (Kovacs et al. Acta Neuropathol. 2020 Aug;140(2):99-119)
- CBD (Dickson et al. J Neuropathol Exp Neurol. 2002 Nov;61(11):935-46)
- AGD staging (Saito et al. J Neuropathol Exp Neurol 2004;63:911-918)
- ARTAG (Kovacs et al. Acta Neuropathol (2016) 131:87–102) (Kovacs et al. Acta Neuropathol Commun. 2018 Jun 11;6(1):50)
- CTE (McKee et al. Brain. 2013 Jan;136(Pt 1):43-64) (Bieniek et al. J Neuropathol Exp Neurol. 2021 Feb 22;80(3):210-219)
