# Supplementary material for: Down Syndrome Biobank Consortium: A perspective
Source: Alzheimers Dement. 2024 Jan 25;20(3):2262–72. doi: 10.1002/alz.13692 (PMC10984425; doi:10.1002/alz.13692)
Supplement: Supplementary file 4 — Supporting Information [file ALZ-20-2262-s002.pdf]

<u>Supplementary dataset 3</u>.

Four separate PDFs demonstrate the DSBC brain donation brochure in English, Hindi, Spanish and Bengali. Plans are to translate this brochure into several more languages and make it available at the DSBC website.

Does my religion prevent brain donation?

Most religions are not against a person donating their brain to research. Even after a brain donation, a person can have an open casket funeral.

If you are wondering more about your religion and brain donation, please go to this web site: https://organdonor.gov/about/donors/religion.html

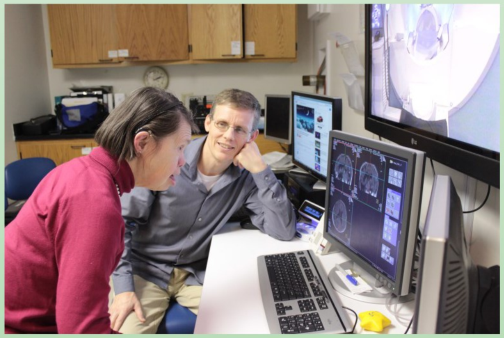

Questions?
Please contact us:

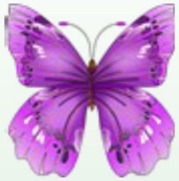

How can I find out more?

To find out more about the Down syndrome Brain Donation program at DSBC, please contact your local health care clinic, hospital, or send inquiries to:

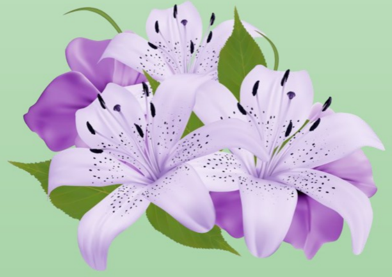

DOWN SYNDROME BIOBANK CONSORTIUM

Facts about Brain Donation

For people with Down syndrome and their families

From the Down Syndrome Biobank Consortium

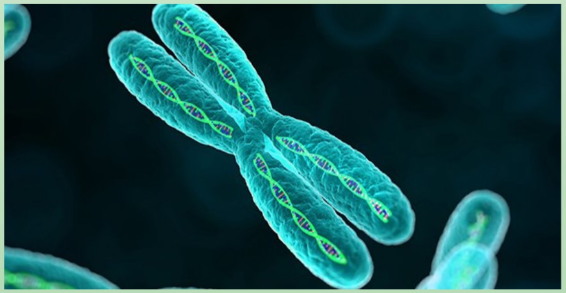

UC Irvine
University of Kentucky
Barrow Neurological Institute
Barcelona Sant Pau Hospital
University of Colorado
New York University
Cambridge University
Karolinska Institutet
Medical University of South Carolina

What is dementia?

Dementia is a brain illness that leads to memory impairment. To understand this illness and come up with new treatment options, researchers need to study the brain. Brain donation improves our understanding of what happens in the brain of someone with Down syndrome (DS), while giving hope to the DS community.

Why is brain tissue needed?

The human brain is complex and difficult to study in living people. Research into conditions that affect the brain has to be done in tissue donated after death. A better understanding is the first key step towards new treatments.

What is involved in brain donation?

An autopsy is the examination of brain tissue after death by a specialist called a pathologist and will be performed as soon as possible AFTER the person has passed. Brain donation does not leave any marks on the hairline or face.

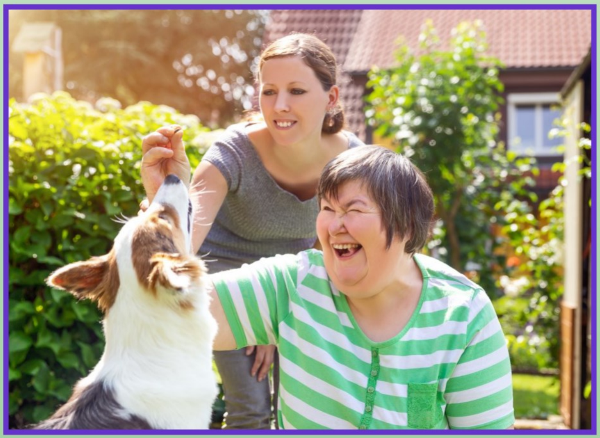

Who can donate?

Because dementia research depends on all types of brain tissue, including those with no obvious disease, anyone over the age of **18** can register to be a donor. A person's next of kin can consent to a brain donation, if the person is unable to do this on their own.

About the DS Biobank consortium

The consortium consists of 12 different research groups who have devoted their research to Down syndrome. By working together, we can unlock the mysteries around trisomy 21 and allow those with this condition to live healthy lives.

¿Mi religión impide la donación de cerebro?

La mayoría de las religiones no se oponen a que una persona done su cerebro para investigación. Incluso después de una donación de cerebro la persona puede tener un funeral con el ataúd abierto.
Si tienes más dudas acerca de tu religión y la donación de cerebro, por favor visita: https://organdonor.gov/about/donors/religion.html

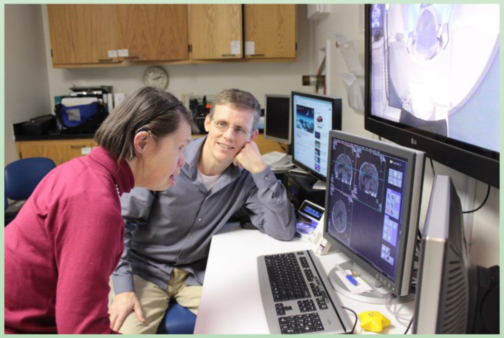

¿Tienes más preguntas?
Contáctanos en:

Lotta.granholm@cuanschutz.edu

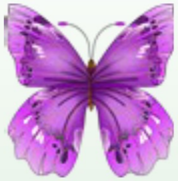

¿Cómo puedo obtener más información?

Para obtener más información sobre el programa de Donación de Cerebro por parte de personas con síndrome de Down en el DSBC, por favor contacta con tu clínica de atención médica local, hospital, o envía tus preguntas a:

https://medschool.cuanschutz.edu/neurosurgery/research-and-innovation/services/down-syndrome-biobank

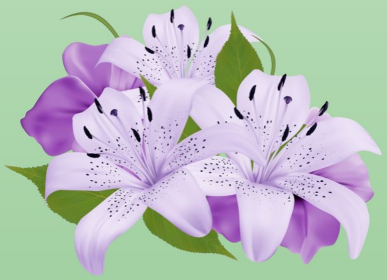

DOWN SYNDROME BIOBANK CONSORTIUM

Datos sobre la donación de cerebro

Para personas con syndrome de Down y sus familias

Del Down Syndrome Biobank Consortium

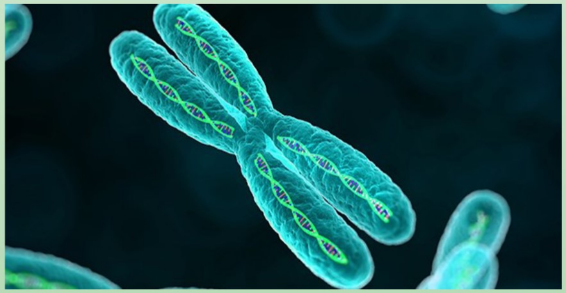

UC Irvine
Barrow Neurological Institute
Barcelona Sant Pau Hospital
Barcelona Clinic Neurological Tissue Bank
University of Colorado
New York University
Cambridge University
London Down Syndrome Consortium
Medical University of South Carolina
University of Calcutta

¿Qué es la demencia?

La demencia es un síntoma asociado con cambios cerebrales que conduce principalmente a la pérdida de memoria. Para comprender esta enfermedad y desarrollar nuevos tratamientos, los investigadores necesitan estudiar el cerebro. La donación de cerebro mejora nuestra comprensión de lo que sucede en el cerebro de una persona con síndrome de Down (SD), al tiempo que brinda esperanza a la comunidad de SD.

¿Por qué se necesita tejido cerebral?

El cerebro humano es complejo y difícil de estudiar en personas vivas. La investigación sobre las enfermedades que afectan al cerebro debe realizarse en tejido donado después del fallecimiento. Una mejor comprensión es el primer paso clave hacia nuevos tratamientos.

¿Qué implica la donación de cerebro?

Una autopsia es el examen del tejido cerebral después del fallecimiento, realizado por un especialista llamado patólogo. Se lleva a cabo lo antes posible DESPUÉS de que la persona haya fallecido. La donación de cerebro no deja marcas en la línea del cabello ni en la cara.

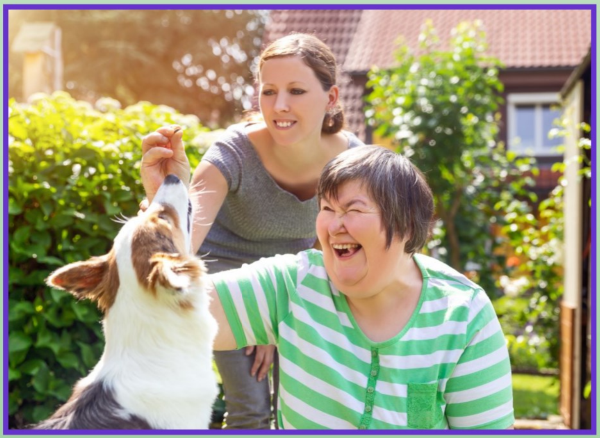

¿Quién puede donar?

Dado que la investigación sobre la demencia requiere de todo tipo de tejido cerebral, incluyendo el de aquellos sin enfermedades evidentes, cualquier persona mayor de 18 años puede registrarse como donante. El familiar más cercano de una persona puede dar su consentimiento para la donación de cerebro si la persona no puede hacerlo por sí misma, aunque deben tenerse en cuenta las leyes locales (consultanos!)

Acerca del DS Biobank Consortium

El consorcio está compuesto por 12 grupos de investigación diferentes que han dedicado sus investigaciones al síndrome de Down. Al trabajar juntos, podemos desvelar los misterios entorno a la trisomía 21 y permitir que aquellos con esta condición lleven vidas saludables.

আমার ধর্মীয় বিশ্বাস কি মরণোত্তর মস্তিষ্ক প্রদানে বাঁধা দেয় ?

অধিকাংশ ধর্মেই গবেষণার জন্য মরণোত্তর মস্তিষ্ক দান করা নিয়ে কোনো আপত্তি নেই। মস্তিষ্ক দান করবার পরেও, একজন মানুষের সসম্মানে অন্ত্যেষ্টিক্রিয়া পালন করায় কোনো সমস্যা হয় না। আপনার ধর্মীয় বিশ্বাস অনুযায়ী মরণোত্তর মস্তিষ্ক দান সম্বন্ধে আরো তথ্যের জন্য এই ওয়েবসাইটটি দেখুন :

https://organdonor.gov/about/donors/religion.html

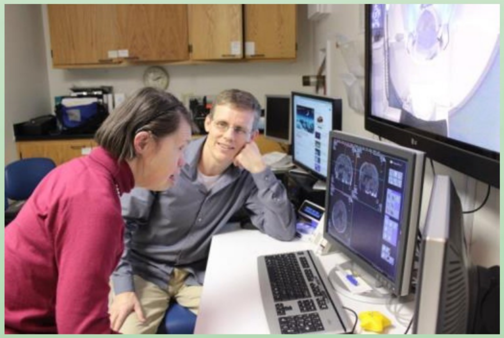

প্রশ্ন থাকলে আমাদের যোগাযোগ করুন

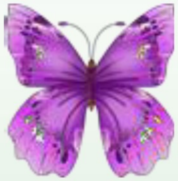

আরো বিশদে কিভাবে জানতে পারি ?

DSBC-র ডাউন সিনড্রোম ব্রেইন ডোনেশন (মরণোত্তর মস্তিষ্ক প্রদান) কর্মসূচি সম্বন্ধে জানবার জন্যে আপনার স্থানীয় চিকিৎসাকেন্দ্র এবং হাসপাতালে যোগাযোগ করতে পারেন

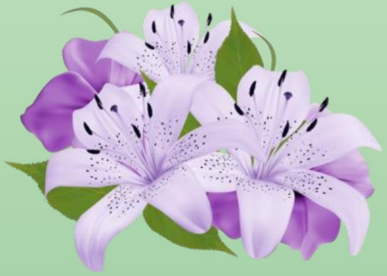

DOWN SYNDROME BIOBANK CONSORTIUM

মরণোত্তর মস্তিষ্ক দান সম্পর্কে তথ্য

ডাউন সিনড্রোম যুক্ত মানুষ এবং তাদের পরিবারের জন্য

ডাউন সিনড্রোম বায়োব্যাংক কনসোর্টিয়ামের পক্ষ থেকে

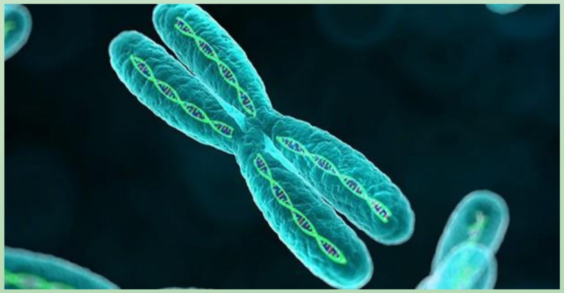

UC Irvine
University of Kentucky
Barrow Neurological Institute
Barcelona Sant Pau Hospital
University of Colorado
New York University
Cambridge University
Karolinska Institutet
Medical University of South Carolina

ডিমেনশিয়া কি ?

ডিমেনশিয়া একটি মস্তিষ্কে-জনিত সমস্যা যার ফলে স্মৃতিশক্তি দুর্বল হয়ে যায়। এই সমস্যাটি কে ভালো করে বোঝবার জন্য এবং উন্নততর চিকিৎসা পদ্ধতি আবিষ্কার করবার জন্যে মস্তিষ্কের গবেষণা খুব জরুরি। একজন ডাউন সিনড্রোম যুক্ত মানুষের মস্তিষ্কের মধ্যে কি ঘটছে সেটির গবেষণার জন্যে মরণোত্তর মস্তিষ্ক প্রদান করা দরকার এবং এই গবেষণার মাধ্যমে ডাউন সিনড্রোম যুক্ত মানুষদের কে আসার আলো দেখানো সম্ভব।

মস্তিষ্কের টিস্যু কেন প্রয়োজন ?

মানুষের মস্তিষ্ক খুবই জটিল এবং তা জীবিত মানুষের মধ্যে অধ্যয়ন করা অসম্ভব।

কি কি কারণের প্রভাবে মস্তিষ্কে সমস্যা হতে পারে তা বোঝার জন্যে গবেষণা প্রয়োজন। উন্নততর চিকিৎসা পদ্ধতি প্রণয়ন করবার জন্যে এই গবেষণাই প্রথম পদক্ষেপ।

মরণোত্তর মস্তিষ্ক প্রদান কিভাবে হয় ?

মৃত্যুর পর যত তাড়াতাড়ি সম্ভব, একজন বিশেষজ্ঞের দ্বারা মস্তিষ্কের টিস্যুর পরীক্ষা করা হয়। এর ফলে মৃত ব্যক্তির চুল বা মুখে কোনো পরিবর্তন হয়না।

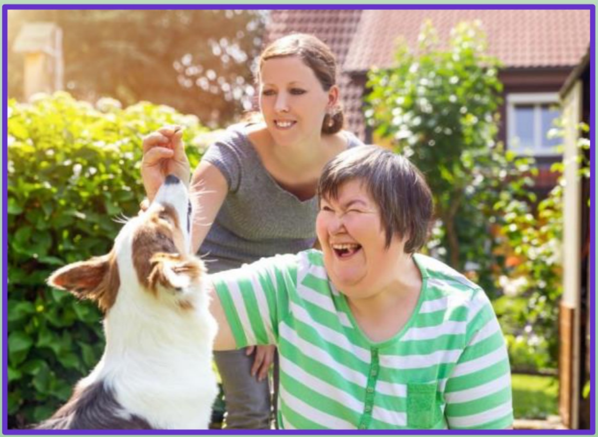

কারা মরণোত্তর মস্তিষ্ক দান করতে পারেন ?

ডিমেনশিয়া সংক্রান্ত গবেষণার জন্যে সবরকম মানুষেরই মস্তিষ্ক টিস্যুর প্রয়োজন, এমনকি যাদের কোনো অসুস্থতা নেই। অতএব, ১৮ বছরের ঊর্ধ্ব বয়স্ক যে কেউ মরণোত্তর মস্তিষ্ক প্রদান করবার জন্যে নাম নথিভুক্ত করতে পারে। যদি কেউ নিজে নথিভুক্ত করতে না পারেন, তাহলে তার নিকটতম আত্মীয় তার হয়ে সম্মতি দিতে পারেন।

ডাউন সিনড্রোম বায়োব্যাংক কনসোর্টিয়াম কি ?

এই কনসোর্টিয়াম-এ ১২টি ডাউন সিনড্রোম বিষয়ক বিজ্ঞানীসমষ্টির দ্বারা গঠিত। আমরা একসাথে কাজ করবার মাধ্যমে ট্রাইসোমি ২১ সম্বন্ধীয় রহস্যগুলো উদ্ঘাটন করতে পারি এবং ডাউন সিন্ড্রোমযুক্ত ব্যক্তিদের জীবনযাপনকে উন্নত করে তুলতে পারি।

क्या मेरा धर्म रोकता है मस्तिष्क दान?

अपना मस्तिष्क दान करने वाले एक व्यक्ति के खिलाफ अधिकांश धर्म नहीं हैं. मस्तिष्क दान के बाद भी व्यक्ति का सम्मानजनक अंतिम संस्कार हो सकता है.

यदि आप अपने धर्म में मस्तिष्क दान के बारे में अधिक जानना चाहते हैं,

कृपया इस वेबसाइट पर जाएँ:

https://organdonor.gov/about/donors/religion.html

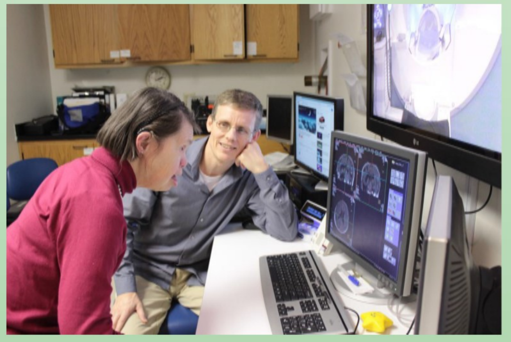

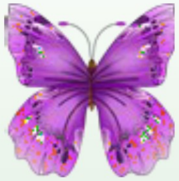

अधिक जानने के लिए..

DSBC में डाउन सिंड्रोम ब्रेन डोनेशन कार्यक्रम के बारे में अधिक जानने के लिए, कृपया अपने स्थानीय स्वास्थ्य चिकित्साकेंद्र, अस्पताल से संपर्क करें।

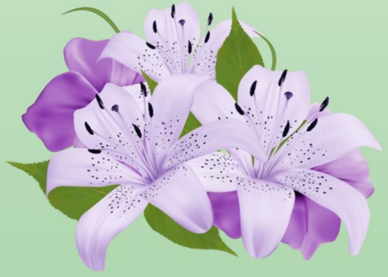

DOWN SYNDROME BIOBANK CONSORTIUM

मस्तिष्क दान के बारे में तथ्य

डाउन सिंड्रोम वाले लोगों और उनके परिवारों के लिए

डाउन सिंड्रोम बायोबैंक कंसोर्टियम

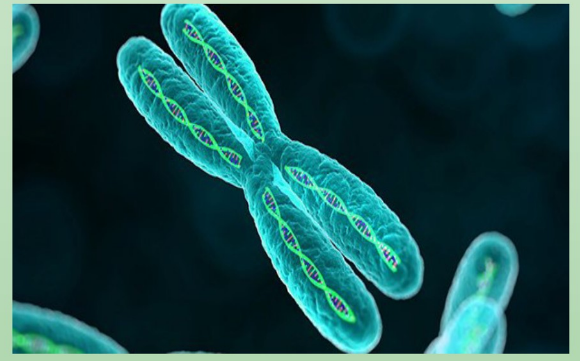

UC Irvine
University of Kentucky
Barrow Neurological Institute
Barcelona Sant Pau Hospital
University of Colorado
New York University
Cambridge University
Karolinska Institutet
Medical University of South Carolina

डिमेंशिया क्या है?

डिमेंशिया एक दिमागी बीमारी है जिसके कारण याददाश्त कमजोर हो जाती है। इस बीमारी को समझने और नए उपचार विकल्पों के साथ आने के लिए दिमाग शोधकर्ताओं को अध्ययन करने की आवश्यकता है। मस्तिष्क दान से हमें डाउन सिंड्रोम व्यक्ति का मस्तिष्क की स्थिति को समझने में मदद मिलती है, और डीएस समुदाय को आशा दे रहे हैं।

मस्तिष्क का आवश्यकता क्यों होता है?

मानव मस्तिष्क जटिल है और जीवित लोगों में इसका अध्ययन करना कठिन है। मस्तिष्क संबंधी स्थितियों का अध्ययन करने के लिए दाताओं की मृत्यु के बाद उनके ऊतक की आवश्यकता होती है। बेहतर समझ हासिल करना नए उपचार विकसित करने की दिशा में महत्वपूर्ण पहला कदम है।

मस्तिष्क दान किस प्रकार से होता है?

एक रोगविज्ञानी विशेषज्ञ द्वारा मृत्यु के बाद मस्तिष्क के ऊतकों की जांच की जाती है और मृत्यु के बाद जितनी जल्दी हो सके शव परीक्षण किया जाएगा। मस्तिष्क दान से हेयरलाइन या चेहरा मैं कोई निशान नहीं पड़ता.

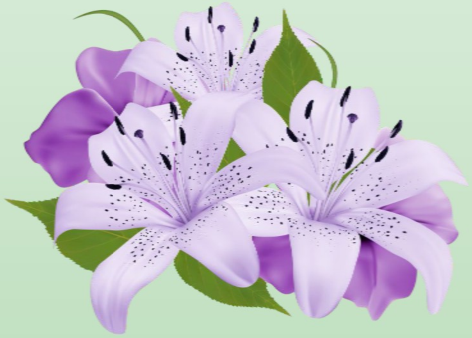

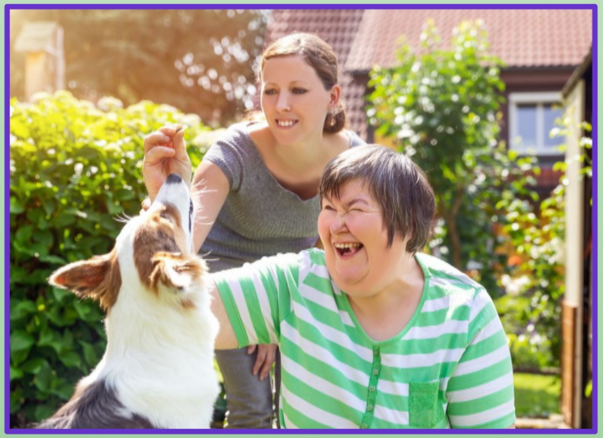

कौन मस्तिष्क दान कर सकता है?

मनोभ्रंश अनुसंधान के लिए मस्तिष्क के विभिन्न ऊतकों की आवश्यकता होती है, यहां तक कि बिना स्पष्ट बीमारियों वाले लोगों से भी। 18 वर्ष से ऊपर का कोई भी व्यक्ति दाता बनने के लिए साइन अप कर सकता है। यदि कोई व्यक्ति स्वयं सहमति नहीं दे सकता है, तो उसका निकटतम परिवार का सदस्य उसके लिए ऐसा कर सकता है।

डीएस के बायोबैंक कंसोर्टियम

कंसोर्टियम में डाउन सिंड्रोम पर केंद्रित 12 अनुसंधान समूह शामिल हैं। सहयोग करके, हम ट्राइसॉमी 21 के रहस्यों को उजागर कर सकते हैं और इस स्थिति वाले व्यक्तियों के जीवन की गुणवत्ता में सुधार कर सकते हैं।
